# Mild Cognitive Impairment: Structural, Metabolical, and Neurophysiological Evidence of a Novel EEG Biomarker

**DOI:** 10.3389/fneur.2015.00152

**Published:** 2015-07-06

**Authors:** Davide Vito Moretti

**Affiliations:** ^1^IRCCS San Giovanni di Dio Fatebenefratelli, Brescia, Italy

**Keywords:** EEG, MRI, SPECT, coherence, memory

## Abstract

**Background:**

Recent studies demonstrate that the alpha3/alpha2 power ratio correlates with cortical atrophy, regional hypoperfusion, and memory impairment in subjects with mild cognitive impairment (MCI).

**Methods:**

Evidences were reviewed in subjects with MCI, who underwent EEG recording, magnetic resonance imaging (MRI) scans, and memory evaluation. Alpha3/alpha2 power ratio (alpha2 8.9–10.9 Hz range; alpha3 10.9–12.9 Hz range), cortical thickness, linear EEG coherence, and memory impairment have been evaluated in a large group of 74 patients. A subset of 27 subjects within the same group also underwent single photon emission computed tomography (SPECT) evaluation.

**Results:**

In MCI subjects with higher EEG upper/low alpha power ratio, a greater temporo-parietal and hippocampal atrophy was found as well as a decrease in regional blood perfusion and memory impairment. In this group, an increase of theta oscillations is associated with a greater interhemispheric coupling between temporal areas.

**Conclusion:**

The increase of alpha3/alpha2 power ratio is a promising novel biomarker in identifying MCI subjects at risk for Alzheimer’s disease.

## Introduction

The mild cognitive impairment (MCI) commonly represents the “at-risk” state of developing dementia. There is, therefore, a need for developing early biomarkers, which allow identifying subjects who could develop the disease, useful for early diagnosis and effective prevention therapies. It is becoming progressively clear that the integration of different biomarkers is a milestone for a correct and early diagnosis of Alzheimer’s disease (AD) (1, 2). To date, the most studied and validated biomarkers are Abeta42 and tau protein in the cerebrospinal fluid (CSF), glucose hypometabolism on fluorodeoxyglucose positron emission tomography (18F-FDG PET), and atrophy of hippocampal volume (HV) on magnetic resonance imaging (MRI) (3, 4). Anyway, some controversies remain to debate. The latter biomarkers have a good sensibility in identifying subjects with neurodegenerative disorders at high risk to convert in dementia, but they lack a reliable specificity that allows a clear-cut diagnosis of the different subtypes of dementias. Of note, in neurodegenerative disorders like AD or other dementias, the brain networks modify many years before clinical manifestations. Recent MRI studies have demonstrated that a large neural network is altered in subjects with prodromal AD (5–10). In particular, subjects with cognitive decline have shown early atrophy and loss of gray matter (GM) in cortical specific brain areas, including precuneus, hippocampal, medial temporal, and parietal lobes (6–8). In the conceptual frame of the integration of biomarkers for an early and highly predictive diagnosis, the EEG could be a reliable tool because it could reflect the activity of cerebral networks (11, 12). Furthermore, EEG linear coherence is a rough but reliable measure of the functional coupling between brain areas (13–16). Recently, it has been demonstrated that the increase of high alpha relative to low alpha power is a reliable EEG marker of hippocampal atrophy (17, 18), temporo-parietal cortical areas atrophy, and lower regional perfusion (19–24). Furthermore, the increase in alpha3/alpha2 frequency power ratio has been demonstrated predictive of conversion of patients with MCI in AD, but not in non-AD dementia (19).

In this study, the evidences about the increase of alpha3/alpha2 power ratio as a promising novel biomarker in identifying MCI subjects at risk for AD were reviewed.

## Materials and Methods

### Subjects

For the present study, 74 subjects with MCI were recruited from the Memory Clinic of the Scientific Institute for Research and Care (IRCCS) of Alzheimer’s and psychiatric disorders “Fatebenefratelli” in Brescia, Italy. All experimental protocols had been approved by the local ethics committee. Informed consent was obtained from all participants or their caregivers, according to the Code of Ethics of the World Medical Association (Declaration of Helsinki).

#### Diagnostic Criteria

Patients were selected from a prospective study on the natural history of cognitive impairment (the translational outpatient memory clinic – TOMC study) carried out in the outpatient facility of the National Institute for the Research and Care of Alzheimer’s Disease (IRCCS, Istituto Centro San Giovanni di Dio Fatebenefratelli, Brescia, Italy). Patients were rated with a series of standardized diagnostic and severity instruments, including the mini-mental state examination (MMSE) (25), the Clinical Dementia Rating Scale [CDRS; (26)], the Hachinski Ischemic Scale [HIS; (27)], the Instrumental and Basic Activities of Daily Living [IADL, BADL; (28)], and a complete neuropsychological assessment (29, 30). All the neuropsychological tests were standardized on Italian population. Thus, scores were compared to normative values with age, education, and gender corrections in an Italian population. In addition, patients underwent diagnostic neuroimaging procedures (MRI) and laboratory testing to rule out other causes of cognitive impairment. Inclusion criteria for the study were all of the following: (i) complaint by the patient, or report by a relative or the general practitioner, of memory or other cognitive disturbances; (ii) MMSE score of 24–27/30, or MMSE of 28 and higher plus low performance (score of 2–6 or higher) on the clock drawing test (29); (iii) sparing of instrumental and basic activities of daily living or functional impairment steadily due to causes other than cognitive impairment, such as physical impairments, sensory loss, gait, or balance disturbances, etc. Exclusion criteria were anyone of the following: (i) patients aged 90 years and older (no minimum age to participate in the study); (ii) history of depression (from mild to moderate or major depression) or juvenile-onset psychosis; (iii) history or neurological signs of major stroke; (iv) other psychiatric diseases, overt dementia, epilepsy, drug addiction, alcohol dependence; (v) use of psychoactive drugs, including acetylcholinesterase inhibitors, or other drugs enhancing brain cognitive functions or biasing EEG activity; (vi) current or previous uncontrolled, or complicated systemic diseases (including diabetes mellitus), or traumatic brain injuries. All subjects were right-handed. These inclusion and exclusion criteria for MCI were based on previous seminal studies (1, 31, 32). Table 1 summarizes demographic and cognitive features of the subjects in the study.

**Table 1:** **Demographic and cognitive characteristics in the whole MCI sample, disaggregated for increased levels of alpha3/alpha2 (means **±** SD for continuous variables or frequency (percentage) for gender and [range] are reported)**.

	Alpha3/Alpha2			
	High	Middle	Low	*p*
**DEMOGRAPHIC AND CLINICAL FUTURES**				
Number of subjects	18	38	18	–
Age (years)	70.4 ± 6.7 [60–85]	68.4 ± 8.2 [52–83]	70.4 ± 7.4 [57–80]	0.55
Sex (female)	13 (%)	24 (%)	14 (%)	0.51
Education (years)	6.6 ± 3.6 [4–18]	7.6 ± 3.7 [3–17]	8.3 ± 4.7 [3–18]	0.42
Mini mental state exam	27 ± 1.7 [23–29]	27.4 ± 1.3 [24–30]	26.9 ± 1.2 [23–30]	0.46
WMHs (mm^3^)	2.78 ± 2.58	5.59 ± 6.60	2.57 ± 2.76	0.09
Alpha3/alpha2	1.29 ± 0.1 [1.17–1.52]	1.08 ± 0.0 [1–1.16]	0.9 ± 0.1 [0.77–0.98]	0.000

*p denotes significance on ANOVA*.

### EEG recordings

The EEG activity was recorded continuously for 5 min from 19 sites by using electrodes set in an elastic cap (Electro-Cap International, Inc.), and positioned according to the 10–20 international systems (Fp1, Fp2, F7, F3, Fz, F4, F8, T3, C3, Cz, C4, T4, T5, P3, Pz, P4, T6, O1, and O2), with the patients in a relaxed condition and closed eyes. The ground electrode was placed in front of Fz. The left and right mastoids served as reference for all electrodes. The recordings were re-referenced off-line to obtain a common average reference. Data were recorded with a band-pass filter of 0.3–70 Hz and digitized at a sampling rate of 250 Hz (BrainAmp, BrainProducts, Germany). Electrodes-skin impedance was set below 5 kHz. Horizontal and vertical eye movements were detected by recording the electrooculogram (EOG). The epochs with ocular, muscular, and other types of artifacts were discarded by two skilled electroencephalographists (15). Moreover, two skilled electroencephalographists checked the data separately and later, they did a common revision. EEG data were reduced off-line in consecutive epochs of 2 s, obtaining a frequency resolution of 0.5 Hz (17, 33–39). For each subject, a number of epochs ranging from 130 to 150 were obtained.

### Analysis of individual frequency bands

A digital FFT-based power spectrum analysis (Welch technique, Hanning windowing function, no phase shift) was computed – ranging from 2 to 45 Hz – obtaining the power density of EEG rhythms with a 0.5 Hz frequency resolution. Two anchor frequencies were selected according to the literature guidelines (16, 19), that is, the theta/alpha transition frequency (TF) and the individual alpha frequency (IAF) peak. IAF and TF were computed for each subject in the study. These anchor frequencies were computed on the power spectra averaged across all recording electrodes. Recent studies have convincingly shown that the IAF is very reliable in rest condition EEG recording (40–46). The TF marks the TF between the theta and alpha bands and represents an estimate of the frequency at which the theta and alpha spectra intersect. TF was computed as the minimum power in the alpha frequency range since our EEG recordings were performed at rest. The IAF represents the frequency with the maximum power peak within the extended alpha range (5–14 Hz). Based on TF and IAF, we estimated the frequency band range for each subject, as follows: delta from TF-4 to TF-2, theta from TF-2 to TF, low alpha band (alpha1 and alpha2) from TF to IAF, and high alpha band (or alpha3) from IAF to IAF + 2. The alpha1 and alpha2 bands were computed for each subject as follows: alpha1 from TF to the middle point of the TF-IAF range and alpha2 from such middle point to the IAF peak. The mean frequency range computed in MCI subjects considered as a whole is: delta 2.9–4.9 Hz; theta 4.9–6.9 Hz; alpha1 6.9–8.9 Hz; alpha2 8.9–10.9 Hz; alpha3 10.9–12.9 Hz; beta1 12.9–19.2 Hz; beta2 19.2–32.4; gamma 32.4–45. Finally, in the frequency bands determined on an individual basis, the relative power spectra for each subject have been computed. The relative power density for each frequency band was computed as the ratio between the absolute power and the mean power spectra from 2 to 45 Hz. The relative band power at each band was defined as the mean of the relative band power for each frequency bin within that band. The alpha3/alpha2 frequency power ratio was computed in all subjects and three groups were obtained according to increasing tertiles values of alpha3/alpha2 power spectra: low (a3/a2 < 1), middle (1 < a3/a2 < 1.16), and high (a3/a2 > 1.17). The tertiles division allows a balanced distribution of the study samples with the advantage to avoid the extreme value in the statistical analysis.

### EEG spectral coherence

EEG coherence represents the covariance of the EEG spectral activity at two electrode locations and can be considered as a measure of temporal synchronization or functional coupling of the EEG signals recorded from pairs of electrodes. (14). Some limitations have to be considered: (1) the linear coherence estimates are affected by volume-conduction of remote EEG sources; (2) coherence analysis just captures the linear component of the functional coupling of the paired EEG oscillations when compared with modern non-linear approaches (15, 16). However, despite the mentioned limitations, the coherence analysis of EEG data is a basic tool available in practically all digital EEG machines used for clinical applications. This is why the analysis of EEG coherence is the most common methodological approach for the study of functional coupling of EEG oscillations in aging (14–16):

$$\text{Coherence }(\text{f})=\frac{|\text{Cross}-\text{spectrum (f) xy}{|}^{2}}{\left(\text{Autospectrum}(\text{f})(\text{x})\right)\left(\text{Autospectrum}(\text{f})(\text{y})\right)}$$

where f denotes the spectral estimate of two EEG signals x and y. The numerator contains the cross-spectrum for x and y (fxy), while the denominator contains the respective autospectra for x (fx) and y (fy). This procedure returns a real number between 0 (no coherence) and 1 (max coherence). The EEG coherence was computed at the fronto-temporal (F3–T3 and F4–T4), temporo-parietal (T3–P3 and T4–P4), and fronto-parietal electrode pairs of interest (F3–P3, F4–P4) to obtain intrahemispheric coherence. Furthermore, EEG coherence was computed at frontal, temporal, and parietal electrode pairs of interest (F3–F4, T3–T4, and P3–P4) to obtain interhemispheric coherence.

Coherence was computed both in MCI subjects and in a group of 70 in normal controls matched for age, sex, and education.

### MRI scans

For each subject, a high-resolution sagittal T1-weighted volumetric MRI scans were acquired at the Neuroradiology Unit of the “Citta` di Brescia” Hospital, Brescia, by using a 1.0 T Philips Gyroscan scanner, with a gradient echo 3D technique: TR = 20 ms, TE = 5 ms, flip angle = 30°, field of view = 220 mm, acquisition matrix 256 × 256, slice thickness 1.3 mm.

### Cortical thickness estimation steps

Cortical thickness measurements for 74 MCI patients were made using a fully automated MRI-based analysis technique: FreeSurfer v5.1.0, a set of software tools for the study of the cortical and subcortical anatomy. Briefly, in the cortical surface stream, the models of the boundary between white matter and cortical gray matter (CGM) as well as the pial surface were constructed. Once these surfaces are known, an array of anatomical measures becomes possible, including cortical thickness, surface area, curvature, and surface normal at each point on the cortex. In addition, a cortical surface-based atlas has been defined based on average folding patterns mapped to a sphere, and surfaces from individuals can be aligned with this atlas with a high-dimensional non-linear registration algorithm. The surface-based pipeline consists of several stages previous described in detail (47).

### Single subject analysis

For each subject, the T1-weighted, anatomical 3-D MRI datasets were converted from Dicom format into .mgz format, then intensity variations are corrected, and a normalized intensity image is created. The volume is registered within the Talairach atlas through an affine registration. Next, the skull is stripped using a deformable template model (48), and extracerebral voxels are removed. The intensity normalized, skull-stripped image is then operated on by a segmentation procedure based on the geometric structure of the gray–white interface. Voxels are classified as white or GM and cutting planes are chosen to separate the hemispheres from each other (49–55).

#### Group Analysis

In order to relate and compare anatomical features across subjects, it is necessary to establish a mapping that specifies a unique correspondence between each location in one brain and the corresponding location in another. Thus, the pial surface of an individual subject is inflated to determine the large-scale folding patterns of the cortex and subsequently transformed into a sphere to minimize metric distortion. The folding patterns of the individual are then aligned with an average folding pattern using a high-resolution surface-based averaging. Thickness measures were mapped to the inflated surface of each participant’s brain reconstruction allowing visualization of data across the entire cortical surface. Finally, cortical thickness was smoothed with a 20-mm full width at half height of Gaussian kernel to reduce local variations in the measurements for further analysis.

## SPECT Scan

About 27 patients and 17 normal controls underwent single photon emission computed tomography (SPECT) scan in the nuclear medicine department of the Ospedali Riuniti, Bergamo. Each subject received an intravenous injection of 925 MBq of technetium-99m ethyl cysteinate dimer (^99m^Tc-ECD) in resting conditions, lying supine with eyes closed in a quiet, dimly lit room. Forty to sixty minutes after injection, brain SPECT was performed using a dual-head rotating gamma camera (GE Elscint Helix) equipped with low energy-high resolution, parallel hole collimators. A 128 × 128 pixel matrix, zoom = 1.5, was used for image acquisition with 120 views over a 360° orbit (in 3° steps) with a pixel size and slice thickness of 2.94 mm. Butterworth filtered-back projection (order = 7, cutoff = 0.45 cycles/cm) was used for image reconstruction, and attenuation correction was performed using Chang’s method (attenuation coefficient = 0.11/cm). Images were exported in DICOM format.

### SPECT processing protocol

To achieve a precise normalization, we generated a study-specific SPECT template using both SPECT and MRI scans of all patients and normal controls under study, following a procedure described in detail elsewhere (56, 57). Briefly, we created a customized high-definition MRI template, we converted SPECT scans to Analyze format using MRIcro, and we coregistered them to their respective MRI scans with SPM2 (SPM, Statistical Parametric Mapping, version 2; London: Functional Imaging Laboratory). We normalized each MRI to the customized MRI template through a non-linear transformation (cut-off 25 mm), and we applied the normalization parameters to the coregistered SPECT. We obtained the customized SPECT template as the mean of all the latter normalized SPECT images. The creation of a study-specific template allows for better normalization, since the low uptake in ventricular structures and the cortical hypoperfusion effects frequently present in elderly patients are accounted for (56, 57).

## Statistical Analysis

### MRI statistical analysis

Differences between groups in sociodemographic and neuropsychological features were analyzed using SPSS version 13.0 (SPSS, Chicago, IL) performing an analysis of variance (ANOVA) for continuous variables and paired χ^2^ test for dichotomous variables. For continuous variables, post hoc pairwise comparisons among groups were performed with the Games-Howell or Bonferroni tests depending on the homogeneity of variance tested with Levene’s test.

Concerning the neuroimaging analysis, the Qdec interface in Freesurfer software was used: a vertex-by-vertex analysis was carried out performing a general linear model to analyze whether any difference in mean cortical thickness existed between groups (low: a3/a2 < 1 μV^2^; middle: 1 < a3/a2 < 1.16 μV^2^; high: a3/a2 > 1.17 μV^2^). The following comparisons were carried out: High vs. Low, High vs. Middle, and Middle vs. Low. Age, sex, education, global cognitive level (MMSE score) were introduced as covariates in the analysis to avoid confounding factors. We first tried to apply an appropriate Bonferroni multiple-comparison correction in our analysis (at *p* < 0.05 corrected). Unfortunately, no *p*-value survived after this correction. Thus, we choose to set a more restrictive significance threshold at *p* < 0.001 uncorrected for multiple comparisons. Moreover, we considered as significant only the clusters, which also were wide equal or major to 30 mm^2^. Finally, a surface map was generated to display the results on an average brain. For illustrative purpose, significance was set to a *p*-value of <0.01 uncorrected for multiple comparisons.

### Memory tests statistical analysis

As a control analysis, in order to exclude casual relationships between EEG markers and cortical volumes, a correlation between brain areas and memory performance has been studied. The correlation analysis was performed on the three samples separately (high, low, and middle a3/a2 frequency power ratio) and the entire sample (high and low plus middle a3/a2 frequency power ratio grouped together). An exploratory analysis of non-linear correlation does not fit into the purpose of testing our a priori hypothesis. Indeed, we choose to apply a measure of linear dependence led by our a priori hypothesis that MCI group with the greater cortical thinning and higher a3/a2 EEG frequency power ratio (indicating an incipient AD) should show a clear correlation with the memory tests performance, in the sense that, even if in the cognitive tests scores there are no significant differences; both an increase in cortical thinning and alpha3/alpha2 EEG frequency power ratio corresponds to a decrease in memory performance and vice versa. The correlation analysis on a vertex-by-vertex basis was performed specifically for the following neuropsychological memory test results: Babcok Test, Rey auditory verbal learning test (AVLT) immediate recall, and Rey AVLT delayed recall. The analysis was thresholded at *p* < 0.001 uncorrected for multiple comparisons while results were mapped at *p*-value of <0.005 uncorrected for illustrative purpose. Only the clusters that survived at the statistical threshold and wide equal to or major than 15 mm^2^ were considered as significant.

### EEG coherence statistical analysis

The EEG coherence session was composed of three ANOVAs for: (1) left intrahemispheric, (2) right intrahemispheric, and (3) interhemispheric coherence. Each ANOVA was a three-way interaction with group as independent variable and electrode pairs and frequency as dependent variables. Greenhouse-Geisser correction and Mauchley’s sphericity tests were applied to all ANOVAs.

### SPECT statistical analysis

All statistical analyses were performed using SPSS software ver. 13.0. We investigated significance of the differences between the two groups (MCI at low and at high risk to develop AD) in socio-demographic, clinical, and cognitive features using χ^2^ test for categorical variables (sex and ApoE carriers) and Student’s independent *t*-test for continuous variables (volumetric, perfusional features, and EEG frequencies). In all cases, we set the significant threshold at *p* < 0.05. Since native SPECT scans were coregistered to their respective MRI images, and the study-specific SPECT template was coregistered to the high-definition MRI template, all the normalized SPECT and MRI images used for the statistical analysis were coregistered to the SPM standard anatomical space. Moreover, Pearson’s *r* correlations were assessed between the selected perfusion ROIs in terms of age-corrected scores (W scores) and the acquired EEG frequencies in both groups.

## Results

### MRI

Table 1 shows the sociodemographic and neuropsychological characteristics of MCI subgroups defined by the tertile values of alpha3/alpha2 frequency power ratio. The ANOVA analysis showed that groups were well-paired for age, sex, white matter hyperintensities (WMHs) burden, education, and global cognitive level. However, age, sex, education, global cognitive level (MMSE score), and WMHs were introduced as covariates in the subsequent analysis to avoid confounding factors. Alpha3/alpha2 frequency power ratio levels were significant at Games-Howell post hoc comparisons (*p* = 0.000). The data of same subjects were used in previously published works of our group (58–60).

**Figure 1:**
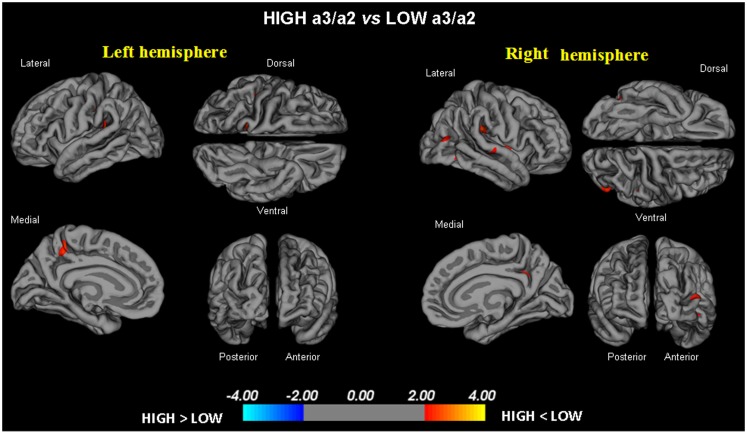
**In red are represented the brain regions with higher regional cortical thickness in MCI with high a3/a2 ratio as compared to MCI with low a3/a2 ratio (*p* < 0.01 uncorrected)**. The color-coding for *p*-values is on a logarithmic scale. Results are presented on the pial cortical surface of brain: dark gray regions represent sulci and light gray regions represent gyri.

**Figure 2:**
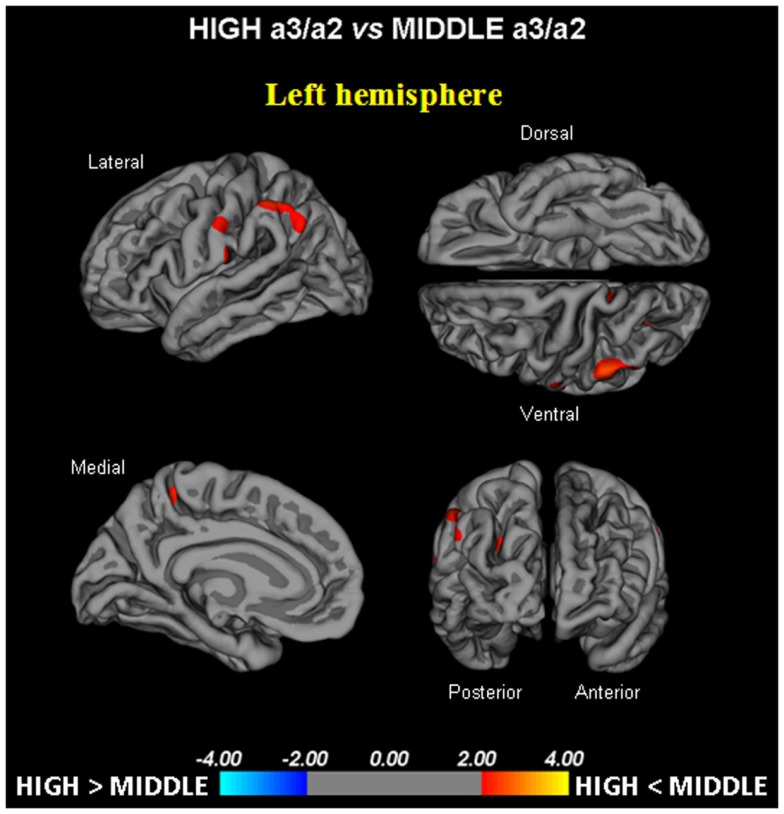
**In red are represented the brain regions with higher regional cortical thickness in MCI with high a3/a2 ratio as compared to MCI with middle a3/a2 ratio (*p* < 0.01 uncorrected)**. The color-coding for *p*-values is on a logarithmic scale. Results are presented on the pial cortical surface of brain: dark gray regions represent sulci and light gray regions represent gyri.

Pattern of cortical thickness between groups:

- High vs. Low: when compared to subjects with low a3/a2 ratios, patients with High a3/a2 ratio show thinning of the bilateral superotemporal, supramarginal, precuneus cortex, and the right inferior parietal and insula. The total CGM reduction in High a3/a2 as compared to low a3/a2 frequency power ratio group was 471 mm^2^ (Figure 1).
- High vs. Middle: The same group showed a similar but less wide pattern of cortical thinning when compared to middle a3/a2 frequency power ratio group: the regions of atrophy were located in the left supramarginal gyrus, left precuneus and postcentral cortex. The total CGM reduction in High a3/a2 as compared to Middle a3/a2 frequency power ratio group was 160 mm^2^ (Figure 2). When High a3/a2 group was compared to Low a3/a2 group, the total extension of cortical thinning (471 mm^2^) was 34% wider than High a3/a2 vs. Middle a3/a2 group (160 mm^2^). No regions of major cortical atrophy were found in groups with Middle or Low a3/a2 power ratio when compared to High a3/a2 group. No significant cortical thickness differences were found between Middle and Low a3/a2 frequency power ratio groups.

#### EEG Coherence Results

The ANOVA results for right intrahemispheric coherence revealed significant interactions between groups, frequency, and electrode pairs (F14, 1596 = 5.54; *p* < 0.0000). Duncan’s post hoc test showed a significant decrease in all frequency on all electrode pairs (all *p*’s < 0.0001) in MCI group. Other comparisons were not significant.

**Figure 3:**
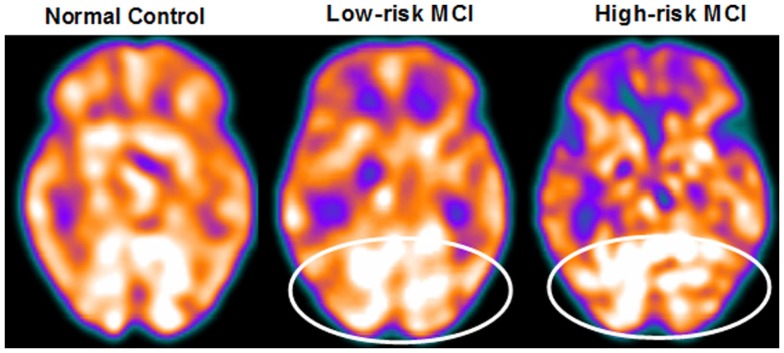
**SPECT visual rating**. The output shows a SPECT visual inspection of glucose uptake metabolism: the white square denotes an area of mild-to-moderate temporo-parietal hypometabolism in one of the 14 at low risk and in one of the 13 at high risk MCI patient respect to one of the 17 enrolled controls. The results are representative of the entire groups.

The ANOVA results for interhemispheric coherence (Figure 3) revealed significant interactions between groups, frequency, and electrode pairs (F14, 1596 = 2.27; *p* < 0.0047). Duncan’s post hoc test showed a significant increase in all frequencies (*p* < 0.05) that was particularly significant in theta frequency on temporo-parietal electrodes (all *p*’s < 0.001) in MCI group. Other comparisons were not significant.

The ANOVA results for left intrahemispheric coherence revealed significant interactions between groups, frequency, and electrode pairs (F14, 1596 = 5.05; *p* < 0.0000). Duncan’s post hoc test showed a significant decrease in all frequency on all electrode pairs (all *p*’s < 0.0001) in MCI group. Other comparisons were not significant.

#### Correlations Between Neuropsychological Memory Tests and Cortical Thickness

Babcock Test: a significant positive correlation was found in High alpha3/alpha2 group between logical memory performance at Babcock test and thickness values in the left caudal middle frontal (cluster size = 36 mm^2^; stereotaxic coordinates *x, y, z* = –34, 22, 47; *r* = 0.80; *p* = 0.0001), left inferior temporal sulcus (15 mm^2^; –54, –28, –26; *r* = 0.72; *p* = 0.001), and right rostral middle frontal cortex (28 mm^2^; 2^3^, 56, –13; *r* = 0.74; *p* = 0.0007). No significant correlation was found with the same regions, or in the other groups, or in the whole sample.

Auditory verbal learning test immediate recall: in High alpha3/alpha2 group, memory performances were significant, related with the cortical thickness values in the bilateral precuneus (left: 47 mm^2^; –21, –61, 20; *r* = 0.78; *p* < 0.0000; right: 58 mm^2^; 20, –60, 25; *r* = 0.72; *p* = 0.0007), left fusiform gyrus (40 mm^2^; –41, –25, –21; *r* = 0.76; *p* = 0.0005), inferior parietal sulcus (43 mm^2^; –46, –60, 11; *r* = 0.74; *p* = 0.0001), inferior temporal sulcus (35 mm^2^; –53, –34, –21; *r* = 0.71; *p* = 0.0008), and right banks of the superior temporal sulcus (44 mm^2^; 48, -48, 9; *r* = 0.81; *p* < 0.000). Memory performance was correlated in the Middle group with both the right precuneus also (*r* = 0.19 and *p* = 0.03), and the right banks of the superior temporal sulcus (*r* = 0.44, *p* = 0.02). No significant associations were found either in the low group or in the entire sample.

Auditory verbal learning test delayed recall: in High alpha3/alpha2 group, memory function correlates significantly with cortical thickness in the bilateral inferior parietal regions (left: 95; –44, –58, 12; *r* = 0.86; *p* < 0.0000; right: 49; 50, –50, 9; *r* = 0.74; *p* = 0.0005), left pericalcarine cortex (54; –7, –8, 11; *r* = 0.76; *p* < 0.0000), superior temporal sulcus (31; –51, –41, –5; *r* = 0.81; *p* = 0.0002), and in the right superior temporal sulcus (22; 56, -34, 13; *r* = 0.73; *p* = 0.001). No significant correlations were found with the same regions, or in the other groups, or in the whole sample.

### SPECT

**Figure 4:**
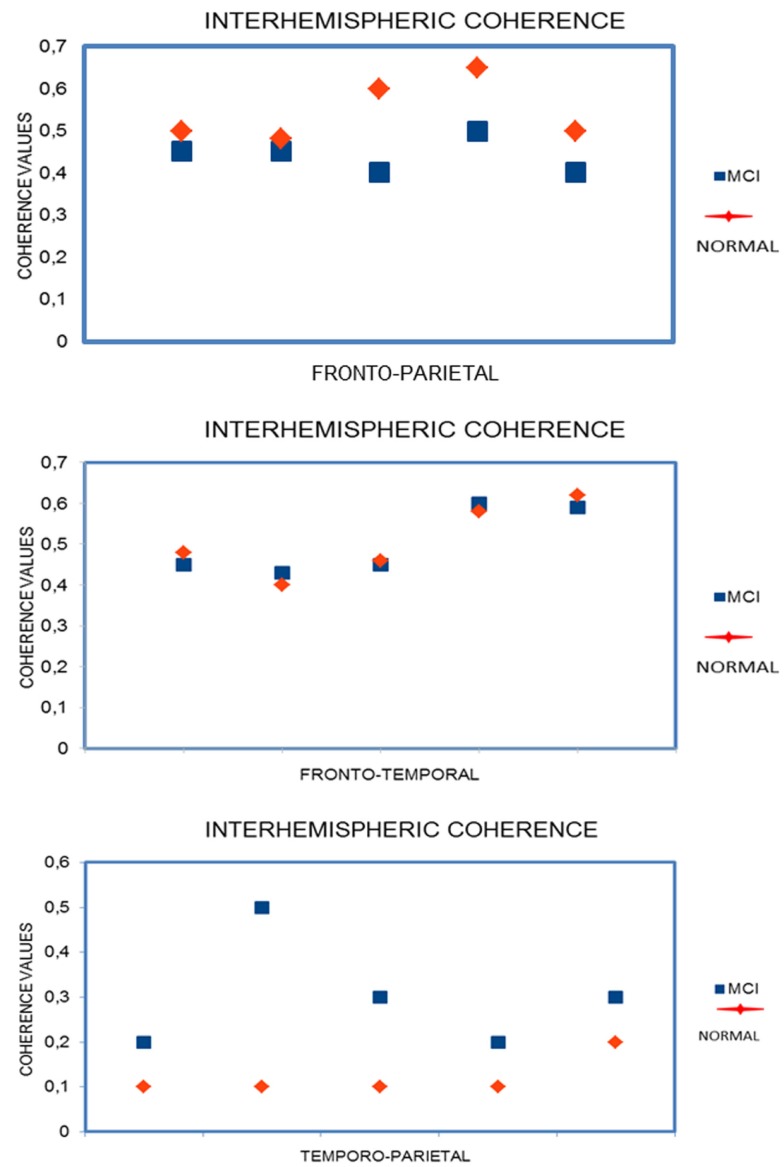
**EEG Coherence statistical results**.

About 27 MCI patients were enrolled in the present study, and they were classified as at high risk (when the a3/a2 frequency power ratio mean was above 1.17) or at low risk (under 1.17) to develop AD. The two groups (AD high risk, *N* = 13; AD low risk, *N* = 14) were similar for age (*p* = 0.56), education in years (*p* = 0.87), gender (*p* = 0.17), ApoE genotype (*p* = 0.15) MMSE scores (*p* = 0.31), and white matter lesions load (*p* = 0.88; Table 2). Figure 4 shows the visual rating scale of the SPECT scans representative of normal control, MCI with low and MCI with high risk to convert in AD, indicating the progressive hypometabolism from normal to high-risk MCI group. Data coming from normal controls were used only to compute W scores in each selected perfusion ROI. Table 3 summarized socio-demographic, clinical, and volumetric features as well as perfusion W scores found in normal controls.

**Table 2:** **Demographic and cognitive characteristics in the MCI sample of the SPECT study disaggregated by increased levels of alpha3/alpha2 (means **±** SD for continuous variables or frequency (percentage) for both gender and ApoE carriers and [range] are reported)**.

	At low-risk MCI	At high-risk MCI	*p*
*N*	14	13	
Age (years) [Range]	69.1 ± 7.6 [57–83]	70.6 ± 5.5 [62–78]	0.555
Gender (females)	6 (43%)	9 (69%)	0.168
Education (years) [Range]	8.2 ± 4.3 [4–18]	7.9 ± 4.5 [3–18]	0.865
MMSE score [Range]	27.9 ± 1.6 [25–30]	27.2 ± 1.9 [24–29]	0.309
ApoE ϵ4 genotype (carriers)	2 (29%)	5 (39%)	0.152
Left hippocampal volume (mm^3^) [Range]	2,606 ± 353 [1,923–3,017]	2,073 ± 412 [1,234–2,641]	0.001
Right hippocampal volume (mm^3^) [Range]	2,581 ± 473 [1,549–3,150]	2,296 ± 501 [1,589–3,086]	141
Wahlund total score [Range]	3.58 ± 3.29 [0.0–10.0]	3.78 ± 2.63 [0.0–7.0]	0.886

*p denotes significance on ANOVA*.

**Table 3:** **Demographic, clinical volumetric, and brain perfusion features of normal elders enrolled in the SPECT study (means **±** SD for continuous variables or frequency (percentage) for both gender and ApoE carriers and [range] are reported)**.

	Normal controls
*N*	17
Age (years) [Range]	69.6 ± 3.2 [65–74]
Gender (females)	9 (53%)
Education (years) [Range]	9.8 ± 4.1 [5–19]
MMSE score [Range]	27.8 ± 1.6 [24–30]
ApoE ϵ4 genotype (carriers)	1/12 (8%)
Left hippocampal volume (mm^3^) [Range]	2,770 ± 274 [2,089–3,351]
Right hippocampal volume (mm^3^) [Range]	2,715 ± 221 [1,881–3,139]
Frontal perfusion (W scores) [Range]	1.2 ± 0.1 [1.1–1.3]
Parietal perfusion (W scores) [Range]	1.4 ± 0.1 [1.3–1.5]
Temporal perfusion (W scores) [Range]	0.4 ± 0.01 [0.4–0.5]
Hippocampal complex perfusion (W scores) [Range]	0.2 ± 0.01 [0.18–0.21]
Thalamic perfusion (W scores) [Range]	0.5 ± 0.02 [0.49–0.57]

*Brain perfusion is always expressed as age-corrected scores (W scores)*.

Analysis of variance results show that the selected cut-off was effective in detecting two different groups: patients with high risk to develop AD show significantly higher alpha3/alpha2 frequency power ratio than patients with low risk (*p* = 0.0001). Moreover, a control analysis was performed on the single frequencies. The results show that the increase of alpha3/alpha2 frequency power ratio was due to both increase of alpha3 (*p* = 0.001) and decrease of alpha2 (*p* = 0.0001) and not to the modification of a single frequency. This control analysis was performed because the change of only one frequency could be due to the chance, but it was not the case.

Of note, no differences were found for theta, beta 1, beta 2, gamma EEG power, and theta/gamma frequency power ratio (all *p* > 0.11). Although the mean perfusion in all the selected ROIs was similar between groups (all *p* > 0.38), in the group with higher alpha3/alpha2 frequency ratio there is a constant trend to a lower perfusion. Moreover, left HVs were smaller for AD-high risk patients than to low risk ones (*p* = 0.001).

In patients at low risk to develop AD, significant Pearson’s *r* negative correlation was found between perfusion in the hippocampal complex and theta rhythm (*r* = –0.544, *p* = 0.044). In patients at high risk to develop AD otherwise, more and dissimilar correlations were found: a positive correlation, inverted respect to patients at low risk, between the perfusion in the hippocampal complex and theta rhythm (*r* = 0.729, *p* = 0.005), while temporal ROI correlated positively with theta/gamma ratio rhythms (*r* = 0.736, *p* = 0.004). No other significant correlations were found in both groups between perfusion ROIs and other EEG rhythms or HVs. Moreover, no significant correlations were found between hippocampal complex ROI and theta rhythm pooling low and high risk patients together (*r* = 0.086, *p* = 0.671).

#### Correlations Between Neuropsychological Memory Tests and Regional Brain Perfusion in High a3/a2 Group and Other Groups

Babcock Test: a significant positive correlation was found in the group with higher alpha3/alpha2 frequency power ratio between logical memory performance at Babcock test, and lower perfusion values in bilateral precuneus (0.63 *p* = 0.03), and superior temporal sulcus (*r* = 0.74, *p* = 0.005). Moreover, a positive correlation was also found with hippocampal atrophy (*r* = 0.75, *p* = 0.001).

Auditory verbal learning test immediate recall: in the group with higher alpha3/alpha2 frequency power ratio, memory performance was significantly related to lower perfusion values in caudal bank of right inferior temporal sulcus and middle frontal gyrus (*r* = 0.75, *p* = 0.003).

Auditory verbal learning test delayed recall: in the group with higher alpha3/alpha2, frequency power ratio memory function correlates significantly with lower perfusion values in the supramarginal gyrus of the inferior parietal lobule (*r* = 0.09, *p* = 0.05).

## Discussion

### Association between EEG markers and GM changes

Our results show that the MCI group with higher alpha3/alpha2 frequency power ratio has a greater global cortical atrophy than the other subgroups, thus confirming a large body of literature (6, 19). Furthermore, the greater atrophy is significant in two specific brain areas: precuneus and supramarginal gyrus (a brain area belonging to the inferior parietal lobule), both in the left and right hemisphere. These results were largely expected considering previous studies. Indeed, structural and functional abnormalities of the precuneus were observed in MCI (61, 62), as well as in AD (63–65), so that the atrophy of precuneus has been considered as a pathognomonic marker of early AD. Of note, recent studies suggest that the precuneus and the posterior cingulate cortex, together with the medial temporal lobe, are selectively vulnerable to early amyloid deposition in AD pathology (66, 67).

### Association between EEG markers and perfusional changes

These results confirm previous studies demonstrating that patients with high risk of developing AD have and reduced SPECT perfusion in temporo-parietal carrefour and inferior parietal lobule (51, 52). Moreover, our results also confirm a well-known correlation between hippocampal atrophy (51). The present study shows a correlation between cerebral perfusion and theta EEG rhythm. However, the correlation emerges only when considering the different groups individuated on the alpha3/alpha2 frequency power ratio. No correlations could be found when the groups are merged. The patients at lower risk to develop AD, who have a constant trend toward a higher brain regional blood perfusion, maintains low levels of theta EEG power while in patients at higher risk, with a basically lower cerebral blood perfusion, theta EEG power tends to be higher. In normal healthy people, theta oscillations are not appreciated in EEG. Anyway, by the analysis of EEG power spectra, a theta power synchronization could be observed over the frontal and temporal areas during learning and memory tasks. The synchronized depolarization of hippocampal neurons produces field potentials that have a main frequency of 3–12 Hz and is usually known as hippocampal theta rhythm (43, 44). The theta rhythms that are recorded during these tasks are thought to be produced by the activation of septal-hippocampal system through the cholinergic innervation originating from the medial septum and the basal forebrain, mediated by and glutamatergic neurons (43, 44). The hyperactivation of the glutamatergic drive, as supposed in the excitotoxic hypothesis of AD, could explain the increase of theta rhythm activity as well as the damage to the information processing observed in AD brain.

### Neurophysiological and clinical implications

Recent studies have demonstrated that, during the successful encoding of new items there is a desynchronization in the temporo-parietal memory-related networks, whereas a synchronization prevents a successful semantic encoding (65, 68). The deleterious role of synchronization has been recently demonstrated by an interesting study facing the intriguing relationship between functional and structural degeneration in AD (66). The authors detected some hub regions (eteromodal associative regions) selectively vulnerable to AD pathology due to the damage of inhibitory interneurons, providing a loss of inhibition at the cellular level. According to the authors, the disinhibition provokes an increasing amount of neural activity at the network level, giving as a result, the hypersynchronization of brain areas. Of note, this overactivity is excitotoxic and determines cellular apoptosis and brain atrophy. In addition, Palop and Mucke emphasize the role of the inhibitory interneuron dysfunction, leading to hypersynchronization (69–72). Our results are in-line with these previous influential studies. A possible integrative view of all the results could be as follows: (1) the higher neuronal activity in the hub regions starts from a disfunction of cellular inhibition; (2) the consequent disinhibition drives neural network to an oversynchronization; (3) this oversynchronization is peculiar to the hub regions with higher amyloid burden; (4) these overactivated regions are prone to degeneration and atrophy; (5) a possible neurophysiologic sign of this oversynchronization is the increase of the alpha3/alpha2 frequency power ratio we have found in typical hub regions (73–76). It is of great interest that there is an overlapping of the brain regions associated with increase of EEG alpha3/alpha2 frequency power ratio (hypersynchronization of upper alpha) in our study and the regions associated with higher amyloid burden related to memory processes (69, 70). Moreover, in the present study, there is a very interesting result. The atrophy of precuneus is coupled with the atrophy in the supramarginal gyrus and, at a lesser extent, with inferior parietal, insula, and superior temporal gyrus. This atrophy pattern is clearly expressed in the group of MCI subjects with higher alpha3/alpha2 frequency power ratio. This finding fits well with the results of a recent study (77), investigating the functional connectivity of human precuneus by resting state fMRI. The authors found that there is a preferential pathway of connectivity of the dorsal precuneus with the supramarginal gyrus, parietal cortex, superior temporal gyrus, and insula. Therefore, the atrophy we detected in the MCI group with higher alpha3/alpha2 frequency power ratio could be hypothesized as the loss of GM in an entire anatomo-functional network more than atrophy of isolated brain areas. Of note, it is widely accepted that AD is the result of a cortical network impairment more than the atrophy of single cortical areas (78). In subjects with low or middle alpha3/alpha2 power ratio, the cognitive impairment is possibly due to cerebro-vascular impairment or non-AD degenerative process, as it happens in the clinical practice.

### Memory performance

In order to exclude a random relationship between EEG marker and cortical atrophy, the correlation between brain areas and the performance on memory tests was investigated in all MCI subgroups. The memory tests were chosen because of their well-known greater impairment in MCI subjects who will convert to AD (1, 9). Our results show no significant memory difference among the groups. This outcome could be apparently paradoxical. Anyway, it should not be considered a very surprising result given that all the patients have a MCI in the early phase of disease, the memory performances did not emerge in a clear way in the first-line memory performance evaluation. On the contrary, when selected groups are obtained, the differences in memory performances could emerge. In other words, when considering the memory performance strictly, the groups are not different. It is a very common situation in clinical practice. Anyway, despite no significant differences in the memory test scores, when focusing on the relationship between the memory performance and a reliable structural marker, such as the cortical thickness, the MCI group with the higher alpha3/alpha2 frequency power ratio showed a negative correlation between memory tests performance and the cortical thickness, as expected in patients with probable prodromal AD. This result confirms the peculiar nature of this MCI group, showing a clear specificity with regard to both the cortical atrophy and the correlated memory performance. Moreover, no other socio-demographical or structural differences were observed in the MCI groups that could explain the correlation analysis results. The cortical areas associated with cortical thinning and those correlated with memory tests performance are only partly overlapping. This could be due to a particular nature of the memory domain, underpinning a large number of brain areas. On the other hand, MCI subjects more susceptible to convert to AD could show impairment also in another cognitive domain like as visuospatial attention or in execution and preparation of spatially guided behavior (79–82). Of note, the cortical network encompassing precuneus and inferior parietal cortex is deeply involved in visuospatial abilities (77). As a speculative interpretation, we could hypothesize that the memory deficits could be due to impaired network underlying the semantic coding of the spatial features of the episodic memory traces. In this view, the atrophy of a specific brain network (more than global volume measures) is more reliable in detecting MCI subjects with prodromal AD. Anyway, the discussion of memory-related brain networks was beyond the scope of the present study. Only a weak negative correlation was found in the middle alpha3/alpha2 EEG power ratio, suggesting a possible degenerative nature of the memory impairment in this group. No significant associations were found in low alpha3/alpha2 power ratio group and the whole sample. Taken together, these results strengthen the position of the MCI group with higher alpha3/alpha2 frequency power ratio as the group at major risk to developing AD.

### Alterations of functional coupling of temporo-parietal areas

Linear EEG coherence is a reliable measure of functional coupling of brain areas. The present study demonstrates that MCI subjects show a general decrease of coherence except an increase of interhemispheric coherence on temporo-parietal regions especially in theta frequency. A previous study has demonstrated that this increase is due to hippocampal atrophy (18).

Previous studies show that the increase of coherence between temporal regions is determined by an increase of excitability (83–87). This hypothesis could receive a support from studies demonstrating a dysregulation of inhibitory GABAergic system following the hippocampal atrophy (87–90). Through the hippocampal commissure, the increase of excitability could spread over the two hemispheres. It could be argued that this activity emerges as a new default mode of brain activity characterized by a hyperexcitability of the cortex, even to a resting state.

### Implications at system level

Klimesch and coworkers have convincingly demonstrated that that the upper alpha band (~10–13 Hz) specifically reflects encoding memory processes (83, 84). Although these authors have found a gain of function associated with the increase of alpha3 power, it should be considered a substantial difference compared to our study. In fact, the increase in alpha3 power was found while performing cognitive tasks, while our results show an increase in of alpha3 power in EEG recording at rest. In a similar manner, also the physiological theta rhythm has different meanings depending on which is considered during a cognitive task (for example, power increase of the theta rhythm during working memory tasks) or at rest, where it is typically a sign of brain injury (for example, in case of cerebral ischemia). This confirms that the study of brain rhythms requires a careful consideration of the cognitive context. Recent EEG and magnetoencefalography (MEG) studies have confirmed that a correct functioning of memory, both in encoding and in retrieval, requires the high alpha rhythm desynchronization (or power decrease) (24, 85–91). The increase in alpha frequency power has been related to the inhibition of cortical brain regions (25, 26, 92). Similarly, the entropy’s theory stated that synchronization is disadvantageous for storing information, as it reduces the flow of information (27). Applying this concept to the neural networks, it has been demonstrated that the degree of information that is encoded in neural assemblies increases as a function of desynchronized and decreases as a function of synchronized firing patterns (77, 93, 94). This hypothesis has been confirmed in clinical studies in patients with memory deficits (95) as well as during states where there is little cognitive processing, e.g., epileptic seizures or slow wave sleep (77, 96, 97). As regarding the cognitive impairment due to AD, it could be hypothesized that the disruption of cortical network due to degenerative disease, inducing synaptic loss and cortical atrophy, could determine an oversynchronization of the brain oscillatory activity. The synchronization state of the high alpha power could prevent the creation of a semantic sensory code and, consequently, of the episodic memory trace (98–101). Of note, in subjects with early cognitive decline, the impairment of the semantic features of memory has been recently accepted as a hallmark for the early AD diagnosis (1, 2). Our results are in line with this hypothesis, suggesting that the increase in power of high alpha brain oscillations reflects a block of the information processes (102–119).

## Study Limitations

Some caveats should be remarked as regards the present study. The results from different previous studies with different methodological design were compared. Any extrapolation has to consider this limitation. However, this does not affect the validity of the conclusions and future prospects that the study opens.

## Conclusion

Increase of upper/low alpha power ratio is associated with cortical thinning, lower perfusion in temporo-parietal areas, memory impairment, and hippocampal atrophy. The increase of alpha3/alpha2 frequency power ratio could be considered as a promising novel biomarker in identifying MCI subjects at risk for developing AD.

## Conflict of Interest Statement

Davide Vito Moretti states that he has no actual or potential conflicts of interest. The author declares that the research was conducted in the absence of any commercial or financial relationships that could be construed as a potential conflict of interest.
